# Transformation of *Anaplasma marginale*

**DOI:** 10.1016/j.vetpar.2009.09.018

**Published:** 2010-02-10

**Authors:** Roderick F. Felsheim, Adela S. Oliva Chávez, Guy H. Palmer, Liliana Crosby, Anthony F. Barbet, Timothy J. Kurtti, Ulrike G. Munderloh

**Affiliations:** a Department of Entomology, University of Minnesota, 1980 Folwell Avenue, St. Paul, MN 55108, United States; b Washington State University, Department of Veterinary Pathology & Microbiology, 402 Bustad Hall, Pullman, WA 99164, United States; c Department of Infectious Diseases and Pathology, College of Veterinary Medicine, 2015 SW 16th Avenue, University of Florida, Gainesville, FL 32610, United States

**Keywords:** Bovine anaplasmosis, *Anaplasma marginale*, Ticks, Genetic transformation, Himar transposase, Homologous recombination

## Abstract

The tick-borne pathogen, *Anaplasma marginale*, has a complex life cycle involving ruminants and ixodid ticks. It causes bovine anaplasmosis, a disease with significant economic impact on cattle farming worldwide. The obligate intracellular growth requirement of the bacteria poses a challenging obstacle to their genetic manipulation, a problem shared with other prokaryotes in the genera *Anaplasma*, *Ehrlichia*, and *Rickettsia*. Following our successful transformation of the human anaplasmosis agent, *A. phagocytophilum*, we produced plasmid constructs (a transposon bearing plasmid, pHimarAm-trTurboGFP-SS, and a transposase expression plasmid, pET28Am-trA7) designed to mediate random insertion of the *TurboGFP* and spectinomycin/streptomycin resistance genes by the *Himar1* allele A7 into the *A. marginale* chromosome. In these trans constructs, expression of the fluorescent and the selectable markers on the transposon, and expression of the transposase are under control of the *A. marginale* tr promoter. Constructs were co-electroporated into *A. marginale* St. Maries purified from tick cell culture, and bacteria incubated for 2 months under selection with a combination of spectinomycin and streptomycin. At that time, ≤1% of tick cells contained colonies of brightly fluorescent *Anaplasma*, which eventually increased to infect about 80–90% of the cells. Cloning of the insertion site in *E. coli* and DNA sequence analyses demonstrated insertion of the entire plasmid pHimarAm-trTurboGFP-SS encoding the transposon in frame into the native tr region of *A. marginale* in an apparent single homologous crossover event not mediated by the transposase. Transformants are fastidious and require longer subculture intervals than wild type *A. marginale*. This result suggests that *A. marginale*, as well as possibly other species of *Anaplasma* and *Ehrlichia*, can be transformed using a strategy of homologous recombination.

## 1 Introduction

Bovine anaplasmosis afflicts cattle from temperate to tropical regions of the world, with disease manifestations that include fever, anemia, abortion, and recurring parasitemic peaks leading to life-long infection (Kieser et al., 1990). Animals older than 2 years are most susceptible to severe disease and death (Rodgers et al., 1994), but economic losses are considerable even in chronically infected cattle, especially in high-yielding dairy breeds and beef cattle due to reduced productivity (Goodger et al., 1979). Today, nearly 100 years after the first description of the causative agent (Theiler, 1910), there are few tools to manage the impact of bovine anaplasmosis. Although tetracycline antibiotics are effective in controlling acute disease (Blouin et al., 2002), treated animals may become chronic carriers and if cured, remain susceptible to reinfection. However, chronic infection imparts protection from clinical disease due to homologous challenge but not heterologous challenge. Similarly, dipping cattle to kill ticks destabilizes an endemic situation by permitting cattle to remain tick-free and thus largely disease-free (Smith et al., 2000), and has additionally accelerated emergence of acaricide resistance. In the event that resistant tick-populations take hold, cattle will be susceptible to anaplasmosis. Considerable efforts have therefore been directed at vaccine development, using live attenuated as well as recombinant vaccines (reviewed in Kocan et al., 2003), none of which have resulted in sterile immunity. These failures have underscored our limited knowledge of the biology of *Anaplasma marginale*, and the need to develop methods for its genetic manipulation. The ability to isolate the agent in continuous culture has removed one of the obstacles, but, as is true for many other obligate intracellular bacteria, genetic manipulation of *A. marginale* has been an elusive goal to achieve. Recent success with a closely related pathogen, *Anaplasma phagocytophilum*, the human granulocytic anaplasmosis agent (Felsheim et al., 2006), has provided renewed incentive to tackle this aim. Here, we describe successful transformation of *A. marginale* to express a fluorescent marker, TurboGFP (Evrogen, Moscow, Russia), and selection of transformants using resistance to spectinomycin and streptomycin.

## 2 Materials and methods

### 2.1 In vitro establishment of *A. marginale* St. Maries

The tick cell line ISE6, derived from embryonated eggs of *Ixodes scapularis* (Munderloh et al., 1994), was propagated at 34 °C in L15B00 as described (Munderloh et al., 1999). The St. Maries isolate of *A. marginale* (Brayton et al., 2005) was established in ISE6 cells from blood of an experimentally infected calf, and maintained by serial passage using medium additionally supplemented with 25 mM HEPES and 0.25% NaHCO_3_, pH 7.6 (Munderloh et al., 1999). Briefly, two 10 months old calves were infected by i.v. inoculation of blood stabilate (1 ml, 50% parasitemia). When the parasitemia had risen to 30% (1 week pi), blood was drawn by aseptic venipuncture from the jugular vein, and 0.5 ml of whole, anticoagulated blood (EDTA–Na_2_) from each calf was added to a confluent, 25-cm^2^ culture of ISE6 cells. The medium was replaced twice weekly until infection was evident by phase contrast microscopy of the live cultures and detection of *A. marginale* colonies in methanol-fixed, Giemsa-stained cells. To identify the isolate as *A. marginale* St. Maries, a 544 bp portion of the *msp1a* gene (nucleotide position 483674–484217 in the *A. marginale* St. Maries genome; GenBank accession Nr. NC_004842; Brayton et al., 2005) was amplified using primers msp1a forward and reverse (Table 1) as described (Bowie et al., 2002). Products were sequenced at the University of Florida DNA Sequencing Core Laboratory (Gainesville) by using an ABI 373 Stretch DNA sequencer (Applied Biosystems, Foster City, CA). Resulting sequences were blasted against the *A. marginale* St. Maries genome (Brayton et al., 2005).

### 2.2 Preparation of host cell-free *Anaplasma*

Bacteria were harvested when the isolate had undergone 12 serial passages in vitro, from an ISE6 culture in which ≥90% of the cells were infected as determined by light microscopic observation of Giemsa-stained cells. Cells in complete medium were suspended by vigorous shaking, and repeatedly sucked through a 27-gauge needle attached to a 5-ml Luer-Lock syringe. *Anaplasma* were separated from cell debris by filtration of the resultant suspension through a 2 μm pore-size filter (Whatman, Florham Park, NJ), and collected by centrifugation at 11,000 × *g* for 11 min at 4 °C. They were immediately processed for electroporation and kept on ice between washes.

### 2.3 Transformation conditions

**Fig. 1:**
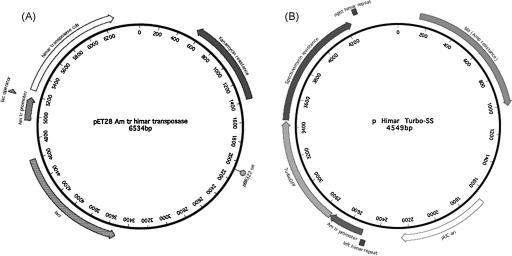
Plasmids used for transformation of *A. marginale* St. Maries. (Panel A) Plasmid pET28 Am tr Himar transposase encoding the A7 allele of *himar1*, driven by the *A. marginale* transcriptional regulator promoter, tr. (Panel B) Plasmid pHimar Turbo-SS encoding the TurboGFP gene and the spectinomycin–streptomycin resistance gene, both driven by the tr promoter. These constitute the transposon that is flanked by the left and right Himar repeats which are recognized by the transposase.

The bacteria were washed twice in ice-cold 250 mM sucrose by centrifugation as above, and finally resuspended in 100 μl of cold 250 mM sucrose with 1 μg each of two plasmids, one (pET28 Am tr A7 himar transposase) encoding the *himar1* allele A7 gene (Lampe et al., 1999) driven by an optimized *A. marginale* tr promoter inserted in the pET28 vector (Novagen, Madison, WI) which contains the *lacIq* gene and *lac* operator to suppress expression of transposase during replication in *E. coli* (Felsheim et al., 2006) (Fig. 1A). The second plasmid carried the transposon encoding the *TurboGFP* gene (Evrogen, Moscow, Russia) upstream of the spectinomycin–streptomycin resistance gene (*aadA*), both driven by the Am tr promoter as described (Felsheim et al., 2006) (Fig. 1B). The region spanning the Am tr promoter and the *TurboGFP* and spectinomycin resistance genes was flanked by repeat sequences recognized by the Himar1 transposase (pHimarAm-trTurboGFP-SS, short designation: pHimar Turbo-SS). Both plasmids were replicated in the methylation deficient *E. coli* mutant GM2163 (New England Biolabs, Beverly, MA), and purified using the endonuclease free Maxi-prep kit from Qiagen (Valencia, CA).

Purified *A. marginale*, approximately 5 × 10^9^ from one 25-cm^2^ culture, were transferred to a 0.2 cm gap electroporation cuvette (Bio-Rad, Hercules, CA), and electroporated at 2.5 kV, 25 μF and 400 Ω, ∼5 ms. They were immediately diluted in 0.5 ml complete medium and overlaid onto a confluent layer of ISE6 cells (comprising approximately 1 × 10^7^ cells) for 30 min at room temperature. Subsequently, the volume was topped to 5 ml, and the culture incubated at 34 °C. Three days after electroporation, spectinomycin (Sigma, St. Louis, MO) was added at a concentration of 100 μg/ml, and the culture fed twice weekly with medium containing spectinomycin. As needed, streptomycin (Sigma) was also included at a concentration of 100 μg/ml. Host cells were not subcultured until after detection of transformants by fluorescence microscopy.

### 2.4 Detection of transformants

Each week, the culture was examined microscopically for the appearance of green fluorescent intracellular anaplasma colonies using a Nikon Diaphot (Nikon, Melville, NJ) equipped for epifluorescence and an EN-GFP filter cube (Chroma Technology Corp., Rockingham, VT) at 200–400× magnification. Alternatively, a drop of live cell suspension under a cover slip was viewed on a Nikon Eclipse E400 at 1000× magnification using a dual (FITC/TRTC) UV light filter set (Chroma). Images were acquired with a Nikon DMX 1200 digital camera and a Dell Optiplex GX 240 computer running Act1 Software (Nikon).

### 2.5 Western blot analysis

To confirm production of TurboGFP, protein was extracted from 2 μm pore-size filter-purified transformed *A. marginale* (AmStMTSS) suspended in sample buffer (0.1 M Tris buffer with 2% SDS, 660 mM β-mercaptoethanol and 0.05% bromophenol blue) and boiled for 5 min. Negative controls were protein extracted from uninfected ISE6 cells. Protein concentration was measured using the RC DC protein assay from Bio-Rad in a BioPhotometer (Eppendorf, Hamburg, Germany). Protein samples (30 ng per well) and 7 μl of SeeBlue^®^ plus 2 protein size markers (Invitrogen, Carlsbad, CA) were resolved in 8–16% SDS-polyacrylamide gradient gels (ISC BioExpress, Kaysville, Utah) by electrophoresis in Tris–glycine buffer for 1.5 h at 100 V. The gel was blotted onto an Immobilon-P membrane (Millipore, Bedford, MA) at 70 V for 2 h, and blocked with 5% nonfat dry milk in PBS at 4 °C overnight. The blot was incubated with TurboGFP specific antibodies (Evrogen) diluted in PBS with 3% bovine serum albumin overnight at 4 °C, washed four times in PBS, and labeled with anti-rabbit IgG conjugated to horseradish peroxidase. Blots were developed with the ImmunoPure^®^ metal enhanced DAB substrate (Pierce, Rockford, IL) system.

### 2.6 Molecular characterization of transformants and insertion site mapping—PCR of fluorescent and resistance markers

To verify and confirm presence of the fluorescence and selection markers in transformed populations of *A. marginale*, bacteria were purified from cultures in which ≥50% of tick cells appeared to contain fluorescent colonies as determined by live microscopy, using the filtration procedure described above. DNA was extracted and purified with the PureGene kit (Gentra Systems, Minneapolis, MN), and used for PCR. A 710 bp portion of the *TurboGFP* gene was amplified using primers Turbo 5′ CON PCR (all primer sequences are listed in Table 1) and Turbo 3′ CON PCR. Primers T-SS CON PCR FOR and T-SS CON PCR were used to amplify a 629 bp portion of sequence starting within the *TurboGFP* gene and extending into the streptomycin–spectinomycin resistance gene *aadA*, using a Techne-312 thermocycler (Techne Inc., Princton, NJ). Primers were produced by Integrated DNA Technologies (Coralville, IA). Reaction mixtures contained AmpliTaq Gold DNA polymerase (Applied Biosystems, Foster City, CA; 0.5 units per μl), 0.5 μM of each primer, 2 mM MgCl_2_, 0.2 mM of each oligonucleotide, and 1 μg of template DNA or water (controls). Cycling conditions were as follows: 94 °C for 5 min; 94 °C for 30 s, 52 °C for 30 s, and 74 °C for 45 s for 30 cycles, followed by a final extension at 74 °C for 5 min. Amplicons were electrophoresed on a 0.8% agarose gel and stained with ethidium bromide.

### 2.7 PCR across insert junctions

**Fig. 2:**
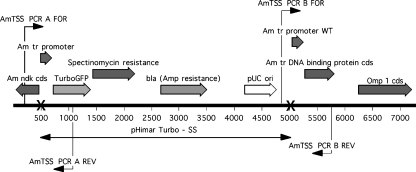
Insertion site map of the *Anaplasma marginale* transformant, AmStMTSS. The two X-marks denote the boundaries of the plasmid integrated in its entirety into the *A. marginale* chromosome at nucleotide position 1026363. Angled arrows indicate the start sites for binding of the primers shown. Primer pair AmTSS PCR A FOR and AmTSS PCR A REV direct the Taq polymerase to read across the upstream junction, whereas primer pair AmTSS PCR B FOR and AmTSS PCR B REV direct reading across the downstream junction between the plasmid and wild type *A. marginale* DNA. PCR using primers AmTSS PCR A FOR and AmTSS PCR B REV direct the polymerase to read across the entire plasmid insert and flanking regions.

The following primers were used: AmTSS PCR A FOR which is complementary to portions of the *A. marginale ndk* gene, and AmTSS PCR A REV which binds to sequences within the *TurboGFP* gene; AmTSS PCR B FOR which anneals to a region just downstream of the *E. coli* origin of replication but still within the plasmid, and AmTSS PCR B REV that is complementary the Am tr DNA binding protein gene (see Fig. 2). The binding sites for these primers are shown in Fig. 2. Products were electrophoresed on 0.8% agarose gels, stained with ethidium bromide, and visualized on a UV light transilluminator.

### 2.8 Insert site mapping

To map the insertion site, transformant DNA was digested with EcoRV (New England Biolabs) for which there are no recognition sites in the plasmid, circularized by ligation with no addition of plasmid vector, and electroporated into *E. coli*. Colonies bearing the insert were selected on SOB plates containing ampicillin 75 μg/ml, spectinomycin 50 μg/ml and streptomycin 50 μg/ml. Plasmids were purified using a mini-prep kit (Roche Molecular Diagnostics, Pleasanton, California) from liquid cultures grown overnight, and cut with EcoRV. Digests were electrophoresed on 0.8% agarose gels, stained with ethidium bromide, and visualized on a UV light transilluminator (data not shown).

### 2.9 Nucleotide sequence analysis

To ascertain and confirm the location and nature of the insert, the DNA from the cloned insert was sequenced using primers AmTSS PCR A FOR and AmTSS PCR B REV. In addition, primers “Turbo up” which binds to the *TurboGFP* gene at position 2939–2960 in pHimar Turbo-SS, and “Spec down” which binds to the *aadA* gene at position 4112–4136 in pHimar Turbo-SS, and standard M13 primers (Promega, Madison, WI) were used to obtain sequence information. Sequencing reactions were carried out at the Advanced Genetic Analysis Center, University of Minnesota using an ABI 377 automated sequencer. To determine the insertion site, nucleotides complementary to primers AmTSS PCR A FOR, AmTSS PCR B REV, “Turbo up” and M13. Forward were trimmed, and the remaining sequences were blasted against the *A. marginale* St. Maries genome (GenBank accession Nr. NC_004842; Brayton et al., 2005).

### 2.10 Real-time PCR assays to determine insert copy number

Real-time quantitative PCR (RT-qPCR) for *opag2* and *TurboGFP* was performed using a DNA Engine Opticon thermal cycler (Bio-Rad). For each 25 μl of reaction, 2 μl of DNA sample was mixed with 12.5 μl of 2× Quantitect Probe PCR master mix (Qiagen), 0.4 μM forward and reverse primers and 0.2 μM probe (MWG Biotech, High Point, NC). The primer sets used were *opag2* forward primer AB1242 and reverse primer AB1243; *TurboGFP* forward primer AB1258 and reverse primer AB1259 (Table 1). The oligonucleotide probe for *opag2*, AB1250 (Table 1), was labeled with 5′ FAM (6-carboxyfluorescein) as a reporter dye, and 3′ TAMRA (6-carboxy-N,N,N′,N′, tetramethylrhodamine) as a quencher (Biosearch Technologies, Novato, CA). For *TurboGFP* the oligonucleotide probe AB1264 (Table 1) was labeled with 5′ TET (6-carboxy-4,7,2′,7′-tetrachlorofluorescein) as a reporter dye, and 3′ BHQ I (Black Hole Quencher I; Biosearch Technologies). Thermocycler conditions used to quantify each gene were 95 °C for 15 min, and 40 cycles of 94 °C for 1 min, 54 °C for 15 s and 60 °C for 1 min. For generation of standard curves, serial dilutions of two different plasmids were used, *opag2*/pCR4-TOPO and pHimar_*msp3*mod. Plasmid *opag2*/pCR4-TOPO contains the *opag2* gene and was used to generate a standard curve for this gene. pHimar_*msp3*mod plasmid contains the full length *TurboGFP* gene and was used to generate a standard curve for *TurboGFP*. Serial dilutions of these plasmids containing 10^8^, 10^7^, 10^6^, 10^5^, 10^4^, 10^3^, 10^2^, and 10^1^ copies were prepared for standard curve calculations. Copy numbers for each sample were calculated based on the standard curves. The *opag2* assay has been described previously (Barbet et al., 2005).

## 3 Results

Amplification of msp1a from the transformant AmStMTSS yielded a 544 bp product that matched the published *A. marginale* St. Maries genome (Brayton et al., 2005) at 100%, and thereby distinguished it from an earlier culture isolate, Virginia (Munderloh et al., 1996).

### 3.1 Live microscopy of fluorescent *A. marginale*

**Fig. 3:**
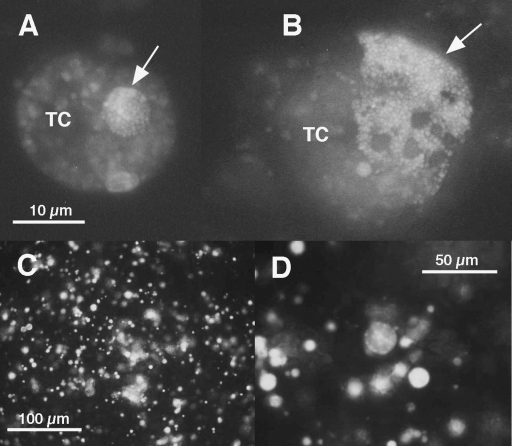
Fluorescence microscopy of live *A. marginale* transformant AmStMTSS. (Panels A and B) Wet mount of cell suspension under a cover slip, viewed with a 100× oil immersion objective. TC, autofluorescent tick host cell; arrows point to AmStMTSS morulae. Magnification is the same in both A and B. (Panels C and D) Live monolayer of ISE6 tick cells infected with transformed *A. marginale* AmStMTSS. (Panel C) Viewed with a 20× objective, panel D viewed with a 40× objective.

Green fluorescent intracellular bacteria forming defined colonies were first detected 2 months post-electroporation in ≤1% of cells in a drop of cell suspension viewed using a Nikon E400 (Fig. 3A and B). Individual bacteria were distinct, and morulae resembled those seen in Giemsa-stained cell preparations (Munderloh et al., 1996). When viewed on the inverted microscope, fluorescent inclusions frequently formed clusters in one or more infected cells (Fig. 3C and D). Transformed *A. marginale* St. Maries were named AmStMTSS, denoting the *Anaplasma* isolate (*A. marginale* St. Maries), GFP species (TurboGFP), and the selectable marker, spectinomycin–streptomycin resistance (SS). When approximately 30% of ISE6 cells contained fluorescent anaplasma, the first subculture was made by dividing the cells into three new flasks with established ISE6 layers as above. Subsequent passages were made every 3–4 weeks when 80–90% of tick cells contained fluorescent morulae by adding 1–2 ml of resuspended cells from an infected culture to a new culture of ISE6 cells. All infected cultures were fed twice weekly by replacing the medium with 5 ml of fresh, complete medium.

### 3.2 Western blots

**Fig. 4:**
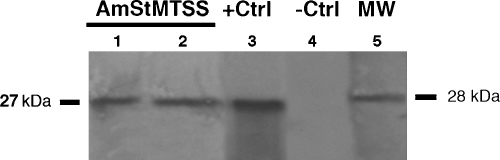
Western blot of protein extracts from transformed *A. marginale* expressing turboGFP (lanes 1 and 2), AmStMTSS; positive control, +Ctrl; lane 3, protein extract of *E. coli* transformed with the plasmid insert cloned from transformant AmStMTSS by EcoRV digestion and recircularization. Negative control, −Ctrl; lane 4, protein extracted from untransformed, wild type *A. marginale* St. Maries. (Lane 5) MW, molecular weight standard.

Polyclonal antibodies to TurboGFP recognized a single band on blots of proteins extracted from transformed AmStMTSS (Fig. 4, lanes 1 and 2), and none in proteins from uninfected ISE6 cells (Fig. 4, lane 4). Likewise, *E. coli* electroporated with circularized DNA recovered from transformed AmStMTSS under antibiotic selection yielded extracts that showed an identical-size band on blots (Fig. 4, lane 3, +Ctrl).

### 3.3 PCR of marker genes

**Fig. 5:**
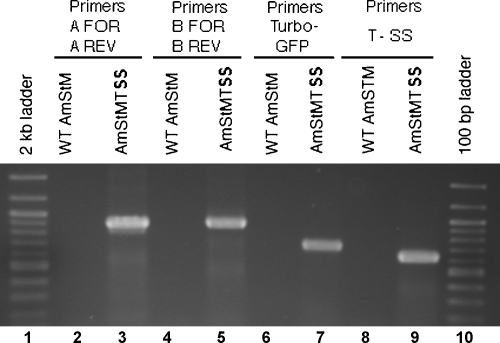
Gel image of PCR results comparing wild type (WT AmStM lanes) and transformed *A. marginale* (AmStMTSS lanes) DNA extracts. (Lane 1) 2-kb ladder; (lanes 2 and 3) DNA amplified using primers AmTSS PCR A FOR and AmTSS PCR A REV (A FOR–A REV). The result confirms integration of the plasmid (pHimar Turbo-SS) downstream from the *A. marginale ndk* gene, coding for nucleoside diphosphate kinase; (lanes 4 and 5) DNA amplified using primers AmTSS PCR B FOR and AmTSS PCR B REV (B FOR–B REV). The result confirms presence of pHimar Turbo-SS sequences downstream from the *E. coli* origin of replication (pUC ori) adjacent to the wild type *A. marginale* tr promoter and the Am tr DNA binding protein gene; (lanes 6 and 7) DNA amplified using primers Turbo 5′ CON PCR and Turbo 3′ CON PCR (TurboGFP) to confirm presence of the TurboGFP gene; (lanes 8 and 9) DNA amplified using primers T-SS CON PCR FOR and T-SS CON PCR REV (T-SS). The result confirms presence of both TurboGFP and resistance gene sequences in the correct position to each other; (lane 10) 100-bp DNA ladder.

Primers specific for portions of the *TurboGFP* gene and the streptomycin–spectinomycin resistance gene *aadA* yielded single bands of the expected size when used in PCR with DNA extracted from transformed anaplasma. Fig. 5 shows an agarose gel with PCR products obtained using *TurboGFP* primers (Fig. 5, lane 7, 710 bp) and *TurboGFP*-*aadA* (T-SS) primers (Fig. 5, lane 9, 629 bp). Likewise, primer pair AmTSS PCR A FOR and AmTSS PCR A REV, designed to amplify DNA spanning the junction between the 5′ region of the insert and wild type *A. marginale* sequences yielded the expected 892 bp product (Fig. 5, lane 3) from AmStMTSS, and primers AmTSS PCR B FOR and AmTSS PCR B REV yielded the correct 908 bp band. No products were obtained in these reactions when DNA from untransformed *A. marginale* was used as template (Fig. 5, WT lanes). Using primers AmTSS PCR A FOR and AmTSS PCR B REV in combination for PCR resulted in amplification of a 1015 bp product from wild type *A. marginale* St. Maries DNA, but a 5564 bp product from AmStMTSS DNA (data not shown) instead of the expected ∼3000 bp band, suggesting insertion of the entire length of the transposon plasmid (pHimar Turbo-SS), not just the transposon itself. Real-time PCR assays of the single copy *opag2* gene gave 1.9 × 10^6^ copies/μl compared to 2.4 × 10^6^ copies/μl of the *TurboGFP* gene or ∼1.26 *TurboGFP* copies/*opag2* copy, which was consistent with approximately a single integrant molecule per genome.

### 3.4 Mapping of the insert region

Initially, spectinomycin–streptomycin resistant clones were obtained from an AmStMTSS genomic library in pLitmus28 as done previously (Felsheim et al., 2006). Primers “Turbo up” and “Spec down” were used to determine the insertion site. The former primer binds near the 5′ end of the *TurboGFP* gene and provided sequence information upstream, extending through the Am tr promoter into the wild type *A. marginale* sequence upstream, as expected. The “Spec down” primer binds to the spectinomycin–streptomycin resistance gene and gave sequence information running downstream into pHimar T-SS vector sequence. This suggested that the entire pHimar T-SS plasmid may have integrated into the AmStMTSS genome, so another genomic library was made without the addition of vector plasmid. All 16 spectinomycin resistant clones yielded plasmid DNA of approximately 7.5 kb following restriction with EcoRV. Primers AmTSS PCR A FOR and AmTSS PCR B REV were then used to sequence portions of the clone recovered from genomic AmStMTSS DNA, yielding about 700 bp of sequence information each that perfectly matched sequences from the regions flanking the insert and extending inward (see Fig. 2). M13 primers Forward and Reverse (Promega, Madison, WI) that bind to plasmid sequence just outside the transposon and read into transposon sequences further confirmed the presence of the entire plasmid pHimar-TSS in the AmStMTSS genome. The size of all PCR products and all sequence data obtained were consistent with the insert map presented in Fig. 2, and support the conclusion that transformation resulted from a single crossover homologous recombination event.

## 4 Discussion

Genetic manipulation of obligate intracellular bacteria poses challenges beyond those ordinarily encountered when working with bacteria that can be grown axenically. The bacteria must be removed from their host cells in order to expose them to the foreign DNA, and any manipulation must not interfere with their capacity to reinfect new host cells. This imposes constraints that combine to reduce the efficiency with which transformants can be successfully recovered. Despite these difficulties, the Himar1 transposase system (Lampe et al., 1999) has thus far been the only method in our hands to consistently achieve transformation of the related pathogen, *Anaplasma phagocytophilum*, albeit at relatively low efficiency when compared to extracellular microbes, e.g., the Lyme disease agent, *Borrelia burgdorferi* (Stewart et al., 2004), in which saturating mutagenesis was achieved. The AmStMTSS transformant described here did not stem from a transposition involving Himar1, but was the result of homologous recombination as a single crossover event at nucleotide position 1026363 in the *A. marginale* St. Maries genome (Brayton et al., 2005). This is similar to the mechanism of transformation described in *Coxiella burnetii* (Suhan et al., 1996). Because we expected transformation to be due to transposase cut and paste activity, the initial sequences using primers “Turbo up” and “Spec down” did not seem to make sense; while “Turbo up” ran into *A. marginale* wild type sequence, “Spec down” yielded downstream vector sequence which indicated that it was neither a transposition event nor plasmid left over from the electroporation. Subsequent sequencing reactions that were carried out with M13 primers demonstrated presence of plasmid downstream from the transposon, and primer AmTSS PCR B REV showed that plasmid sequence was located next to and upstream of the wild type Am tr promoter and the Am tr DNA binding protein gene. Taken together with the sequence results obtained using “Turbo up” and AmTSS PCR A FOR that ran across the junction between the Am tr promoter from the transposon as well as the left himar repeat (Fig. 1) and into the *A. marginale ndk* gene, this showed that the entire pHimar-TSS plasmid had undergone a single crossover homologous recombination event into nucleotide position 1026363 at the start of the wild type Am tr promoter, involving the transposon Am tr promoter. To date, numerous subsequent transformation experiments using constructs and conditions that are successful for *A. phagocytophilum* have not yielded any other *A. marginale* transformants from either the St. Maries or the Virginia isolates which is also in culture in our laboratory (Munderloh et al., 1996). It would be important to define conditions resulting in homologous recombination of selectable markers, yielding gene knock-outs or knock-ins. This would provide a valuable tool for analysis of the function of specific, selected *A. marginale* genes in ticks and cattle.

AmStMTSS appears to be a stable transformant that has been grown in the absence or presence of spectinomycin without losing its fluorescent phenotype for about 10 serial passages. However, it is more fastidious in culture than the wild type parent, and requires longer subculture intervals of ∼3 weeks after a passage involving a 1:5 dilution. Under those conditions, the parent will regrow to ≥90% infection of tick cells within about 10 days. At present, it is not known if the slow growth is related to the presence of the plasmid insert, and/or translation of its additional genetic elements. A transcriptional analysis of AmStMTSS targeting the insert genes could resolve this question. It is conceivable that the two Am tr promoters interfere with each other, or that the *E. coli* origin of replication results in dysfunction or disregulation of normal translational processes. Given the early passage at which AmStMTSS was generated, it is also possible that a slow-growing clone was selected by chance. It will be important to determine if AmStMTSS is infectious for cattle and ticks. If so, it should prove useful for live imaging and pathogenesis studies of bovine anaplasmosis.

## 5 Conclusions

The research presented here has demonstrated that the fluorescent and selectable markers used, genes encoding TurboGFP from a copepod (Evrogen) and spectinomycin–streptomycin resistance, are suitable fluorescent and selectable markers for transformation of *A. marginale*, and are functional in those bacteria. The ability to target specific genes or genomic regions by homologous recombination, whether aiming to introduce foreign genes or generate knock-outs, provides a powerful strategy of genetic manipulation of this important bovine pathogen.

## Conflict of interest statement

None declared.

## Figures and Tables

**Table 1:** Oligo-nucleotides for PCR and sequencing.

Name	Sequence 5′–3′	Target or purpose
msp1a forward	CAT TTC CAT ATA CTG TGC AG	Isolate identification
msp1a reverse	TTG GAG CGC ATC TCT TGC C	Isolate identification
AB1242	AAA ACA GGC TTA CCG CTC CAA	*opag2* forward qRT-PCR
AB1243	GGC GTG TAG CTA GGC TCA AAG T	*opag2* reverse qRT-PCR
AB1258	TCT TCA CCG ACA AGA TCA TCC	TurboGFP forward qRT-PCR
AB1259	GTC CAC CAC GGA GCT GTA GTA	TurboGFP reverse qRT-PCR
AB1250	CTC TCC TCT CGT CAG GGC TCT GCG	*opag2* qRT-PCR probe
AB1264	ATA ACG ATC TGG ATG GCA GC	TurboGFP qRT-PCR probe
Turbo 5′ CON PCR	GGA TAT TAT AAT GGA GAG CGA CGA GAG C	Fluorescent marker
Turbo 3′ CON PCR	CCT ATT CTT CAC CGG CAT CTG CAT C	Fluorescent marker
T-SS CON PCR FOR	CTA CAC CAA CAC CCG CAT CGA GAA G	Selectable marker
T-SS CON PCR REV	CGT TGT TTC ATC AAG CCT TAC GGT C	Selectable marker
AmTSS PCR A FOR	GTT CGT GGC ACC CAT AAC ATC TC	PCR 5′ insert junction sequencing insert
AmTSS PCR A REV	GTG CCC ATC ACC TTG AAG TCG	PCR 5′ insert junction
AmTSS PCR B FOR	TTA GGC ACC CCA GGC TTT ACA C	PCR 3′ insert junction
AmTSS PCR B REV	AAT GCT CGT AGT CAT CCT GCT GC	PCR 3′ insert junction sequencing insert
Turbo up	TAG GTG CCG AAG TGG TAG AAG C	Sequencing insert
Spec down	CAG CCC GTC ATA CTT GAA GCT AGG C	Sequencing insert

## References

[bib1] Barbet A.F., Agnes J.T., Moreland A.L., Lundgren A.M., Alleman A.R., Noh S.M., Brayton K.A., Munderloh U.G., Palmer G.H. (2005). Identification of functional promoters in the *msp2* expression loci of *Anaplasma marginale* and *Anaplasma phagocytophilum*. Gene.

[bib2] Blouin E.F., Kocan K.M., de la Fuente J., Saliki J.T. (2002). Effect of tetracycline on development of *Anaplasma marginale* in cultured *Ixodes scapularis* cells. Vet. Parasitol..

[bib3] Bowie M.V., de la Fuente J., Kocan K.M., Blouin E.F., Barbet A.F. (2002). Conservation of major surface protein 1 genes of *Anaplasma marginale* during cyclic transmission between ticks and cattle. Gene.

[bib4] Brayton K.A., Kappmeyer L.S., Herndon D.R., Dark M.J., Tibbals D.L., Palmer G.H., McGuire T.C., Knowles D.P. (2005). Complete genome sequencing of *Anaplasma marginale* reveals that the surface is skewed to two superfamilies of outer membrane proteins. Proc. Natl. Acad. Sci. U.S.A..

[bib5] Felsheim R.F., Herron M.J., Nelson C.M., Burkhardt N.Y., Barbet A.F., Kurtti T.J., Munderloh U.G. (2006). Transformation of *Anaplasma phagocytophilum*. BMC Biotechnol..

[bib6] Goodger W.J., Carpenter T., Riemann H. (1979). Estimation of economic loss associated with anaplasmosis in California beef cattle. J. Am. Vet. Med. Assoc..

[bib7] Kieser S.T., Eriks I.S., Palmer G.H. (1990). Cyclic rickettsemia during persistent *Anaplasma marginale* infection of cattle. Infect. Immun..

[bib8] Kocan K.M., de la Fuente J., Guglielmone A.A., Meléndez R.D. (2003). Antigens and alternatives for control of *Anaplasma marginale* infection in cattle. Clin. Microbiol. Rev..

[bib9] Lampe D.J., Akerley B.J., Rubin E.J., Mekalanos J., Robertson H.M. (1999). Hyperactive transposase mutants of the *Himar1 mariner* transposon. Proc. Natl. Acad. Sci. U.S.A..

[bib10] Munderloh U.G., Liu Y., Wang M., Chen C., Kurtti T.J. (1994). Establishment, maintenance and description of cell lines from the tick *Ixodes scapularis*. J. Parasitol..

[bib11] Munderloh U.G., Blouin E.F., Kocan K.M., Ge N.-L., Edwards W.L., Kurtti T.J. (1996). Establishment of the tick (Acari: Ixodidae)-borne cattle pathogen *Anaplasma marginale* (Rickettsiales: Anaplasmataceae) in tick cell culture. J. Med. Ent..

[bib12] Munderloh U.G., Jauron S.D., Fingerle V., Leitritz L., Hayes S.F., Hautman J.M., Nelson C.M., Huberty B., Kurtti T.J., Ahlstrand G.G., Greig B., Mellencamp M.A., Goodman J.L. (1999). Invasion and intracellular development of the HGE agent in tick cell culture. J. Clin. Microbiol..

[bib13] Rodgers S.J., Welsh R.D., Stebbins M.E. (1994). Seroprevalence of bovine anaplasmosis in Oklahoma from 1977 to 1991. J. Vet. Diagn. Invest..

[bib14] Smith R.D., Evans D.E., Martins J.R., Ceresér V.H., Correa B.L., Petraccia C., Cardozo H., Solari M.A., Nari A. (2000). Babesiosis (*Babesia bovis*) stability in unstable environments. Ann. N. Y. Acad. Sci..

[bib15] Stewart P.E., Hoff J., Fischer E., Krum J.G., Rosa P.A. (2004). Genome-wide transposon mutagenesis of *Borrelia burgdorferi* for identification of phenotypic mutants. Appl. Environ. Microbiol..

[bib16] Suhan M.L., Chen S.Y., Thompson H.A. (1996). Transformation of *Coxiella burnetii* to ampicillin resistance. J. Bacteriol..

[bib17] Theiler, A., 1910. *Anaplasma marginale* (gen. and spec. nov.). The marginal points in the blood of cattle suffering from a specific disease. In: Report to the Government, Transvaal, South Africa, Veterinary Bacteriology, Dept. of Agriculture 1908-9 (1910), pp. 7–64.

